# Anatomical and Pathological Observations

**Published:** 1845-10

**Authors:** 


BRITISH AND FOREIGN

### MEDICAL REVIEW,

FOR OCTOBER, 1845.

### PART FIRST.

### Analytical and Critical Reviews.

### Art. I.

*Anatomical and Pathological Observations.* By John Goodsir, f.r.s.e., Demonstrator of Anatomy in the University of Edinburgh; and Harry D. S. Goodsir, m.w.s., Conservator of the Museum of the Royal College of Surgeons, Edinburgh.—*Edinburgh,* 1845. 8vo, pp. 128; with Nine Lithographic Plates.

We have had the pleasure, on more than one previous occasion, of drawing the attention of our readers to the meritorious labours of Mr. John Goodsir, whose researches have already gained for him a high rank among the cultivators of the new field, which the achromatic microscope has opened to the anatomist and physiologist; and whose zeal, accuracy, and sagacity give a high promise for the future. It has rarely—we think we may say *never*—been our good fortune to meet with a brochure of the modest pretensions of the one before us, containing so large an amount of original information, put forth in so simple and concise a form. Portions of it have been already presented at various times to the scientific world; but the greater part has been hitherto unpublished; and what has before seen the light has been improved in form, by the extension of the author's views on collateral subjects. He has associated with his own papers, in this publication, some valuable contributions by his brother, Mr. Harry Goodsir, who is zealously devoting himself to a similar line of inquiry; and whose return with the spoils and trophies of the Arctic Expedition on which he has recently proceeded, may be expected some three years hence.

Our sense of the value of the contributions of this "par nobile fratrum" to Anatomical and Pathological *science,* induces us to enter into a more detailed analysis of their Essays, than we should have otherwise thought it necessary to do; considering the general absence, in these memoirs, of any direct practical bearing.

The first paper, entitled "Centres of nutrition," contains what may be called the leading idea of the whole series, which is thus expressed by the author:

"By centres of nutrition I understand certain minute cellular parts existing in the textures and organs. With many of these centres, anatomists have been for some time familiar; but with a few exceptions have looked upon them as embryonic structures. I am inclined to believe in the general existence of such centres, for a certain period at least, in all textures and organs; and to this I wish to direct attention at present.

"The phenomena presented by these centres incline me to regard them as destined to draw from the capillary vessels, or from other sources, the materials of nutrition, and to distribute them by development to each organ or texture after its kind. In this way they are to be considered centres of germination; and I have elsewhere named them germinal spots,—adopting the latter term from the embryologists.

"The centre of nutrition with which we are most familiar is that from which the whole organism derives its origin,—the germinal spot of the ovum. From this all the other centres are derived, either mediately or immediately; and in directions, numbers, and arrangements, which induce the configuration and structure of the being." (p. 1.)

"A nutritive centre, anatomically considered, is merely a cell, the nucleus of which is the permanent source of successive broods of young cells, which from time to time fill the cavity of their parent, [and, carrying with them the cell-wall of the parent] pass off in certain directions, and under various forms, according to the texture or organ of which their parent forms a part.

"There is one form in which nutritive centres are arranged, both in healthy and morbid parts, which is frequently alluded to in the following chapters, and which may be named a germinal membrane. In a germinal membrane, the nutritive or germinal centres are arranged at equal or variable distances, and in certain directions, in the substance of a fine transparent membrane. A germinal membrane is occasionally found to break up into portions of equal size, each of which contains one of the germinal centres. From this it is perceived that a germinal membrane consists of cells with their cavities flattened, so that their walls form the membrane by cohering at their edges, and their nuclei remain in its substance as the germinal centres." (pp. 2-3.)

We have ourselves been gradually led to similar views; of which our readers will find indications in our critique upon Mr. Addison's Researches in our last Number, (p. 124.) And in particular regarding the "germinal membrane" of Mr. Goodsir, which is the same thing with the "basement membrane" of Mr. Bowman, we may state that we have, in common with the former observer, frequently observed indications of nuclei and even of cells in some parts of its substance, whilst other portions, even of the same continuous layer, appeared perfectly homogeneous, as described by the latter. Hence we have little hesitation in assenting to Mr. Goodsir's opinion, that this membrane must be regarded as furnishing the germs of the epithelial cells, which lie upon its surface, or which fill the cavities of the tubuli or cæcal follicles which it forms. In fact, we do not see where else the germs of these cells *can* come from, if they are formed from germs at all; since it is impossible that the cells which are being continually thrown off from the superficial layers should be the parents of the new ones that are being developed in the deepest, and that are springing (as it were) from the surface of the germinal or basement membrane.—In the second Essay, "On the structure and functions of the intestinal villi," we meet with the following additional hints, in regard to the origin of the epithelium from the germinal membrane:

"The germinal membrane, which not only forms the outer membrane of the follicles, under the epithelia, but also the underlying membrane of the villi, contains in its substance germinal centres of an oval form, situated at pretty regular distances. From these the epithelium appears to be reproduced during the intervals of absorption, as stated in the first chapter. During this process of development, the primary membrane appears to split into two laminæ, the epithelia passing out from its nuclei between these. This would account for the epithelia, particularly the prismatic and conical, adhering by their free extremities." (p. 10.)

We believe it would be the most correct to say, that the basement or germinal membrane is continually giving origin, beneath its free or most superficial layer, to new crops of epithelium-cells; whilst it is as continually being renewed, at its attached surface, from the materials supplied by the blood.

With the main subject of the second Essay, we have made our readers acquainted on a former occasion; and we need here only state, that the author has to our minds most successfully proved, that the act of selective absorption is to be attributed to the development of certain cells situated in extremities of the villi, which draw into themselves by their own growth the materials they are afterwards to yield to the absorbent vessels. It has amused us not a little to read discussions in the French Academy, as to the question of priority in the announcement of a very imperfect idea of a somewhat similar kind; both claimants seeming to be equally ignorant, that Mr. Goodsir had published a much more complete account of the process, some months anteriorly to the earliest period specified by themselves.

In the third Essay, on "Absorption, ulceration, and the structures engaged in these processes," Mr. Goodsir has applied the doctrines of cell-development to the pathological phenomena included under these heads. In the study of the normal processes of absorption, nutrition, and secretion, we have had to transfer our attention from the vessels, which simply convey the materials, to the non-vascular portions of the tissues, which are really the active agents in the conversion and application of these materials. We still, however, as Mr. Goodsir remarks, retain in full force our old belief in the active absorbing powers of the vessels, and in the agency of the capillary and lymphatic vessels, in removing parts and modelling the forms. He does not yet attempt to give a complete elucidation of the subject, on the basis of the views just alluded to; but contents himself with adducing certain facts, which plainly indicate the path to be pursued in working out the inquiry. These few facts are, to our minds, however, of the highest interest, and most pregnant character; and we cannot hesitate in transferring them to our pages:

"A rapidly-extending ulcerated surface appears as if the textures were scooped out by a sharp instrument. The textures are separated from the external medium by a thin film. This film is cellular in its constitution, and so far it is analogous to the epidermis or epithelium. It is a peculiarly endowed cellular layer, which takes up progressively the place of the subjacent textures, these being prepared for dissolution, either by the state of the system, the condition of the part, or by some influence induced by the contiguity of the new formation. Carrying out, therefore, the principles at present regarded as regulating the reciprocal functions of textures and vessels, the subjacent textures disappear in consequence of a disturbance of their own forces, consequent upon the appearance of new forces residing in the cellular layer. The disturbance and gradual annihilation of the natural forces residing in the subjacent textures, is indicated by the gradual disappearance of these. That new forces, not formerly existing in the part, are developed, appears from the formation of the cells of the cellular layer. As these appear in rapid succession, and disappear as rapidly, the subjacent textures also disappear, either by previous solution and subsequent absorption by the properties and powers of the former; or under the peculiar circumstances of inflammatory action by the more vigorous growth of the former, monopolizing the resources of the part, the latter dissolving and disappearing by the usual channels of the returning circulation, more rapidly, but according to ordinary laws.

"From this view of the process, it appears that so far from consisting in a diminution of the formative powers of the part, such a progressive ulceration is actually an increase of it. The apparent diminution is a consequence of the extremely limited duration of existence of the cells of the absorbent layer, which die as rapidly as they are formed, disappearing after dissolution, partly as a discharge from the surface, but principally through the natural channels by which the debris of parts which have already performed their allotted functions, are taken up into the organism." (p. 15.)

By this view of the processes of ulceration, we substitute, as Mr. Goodsir very justly remarks, for the hypothetical or aggressive power of absorption ascribed to the veins and the lymphatics, a power which is known to exist in the organic cell during the progress of its growth; and we limit the function of the vessels to that which alone they are known to exert, in health as in disease,—namely, the removal of the effete matter actually set free from the tissues. The general doctrine here upheld derives great support from Mr. Goodsir's observations upon the process of ulceration in bone and articular cartilage:

"When a portion of dead or dying bone is about to be separated from the living, the Haversian canals which immediately bound it are enlarged contemporaneously with the filling of their cavities with a cellular growth. As this proceeds, contiguous canals are thrown into one another. At last, the dead or dying bone is connected to the living by the cellular mass alone. It is now loose, and has become so in consequence of the cellular layer which surrounds it presenting a free surface, and throwing off pus.—In this process, the veins and absorbents act on the osseous texture of the walls of the Haversian canals in no other way than in the natural state of the part. They are mediate, not immediate instruments of absorption. It is the cells of the newly-formed cellular mass, contained in the Haversian canals, which are the immediate cause of the removal of the bone, either by taking it up as nourishment, and substituting themselves in its stead; the bone being prepared for this absorption in a manner analogous to that which occurs in the digestion of food previously to absorption of it by the cells of the gut; or by the active formation of the cells in the new substance monopolizing the resources of the part, and so inducing the disappearance of the osseous texture by the natural channels of the returning circulation.—The process by which a slough in the soft parts is separated from the living textures, is similar to that which occurs in bone." (p. 16.)

Considering the really extra-vascular nature of the ultimate elements of the osseous tissue, it appears to us that the process of absorption must be essentially the same in bone as it is in those shells of mollusca and crustacea, in which a removal of matter was long since proved to take place in certain cases. In both cases it must be a purely *superficial* action; but the surface is extended, by the peculiar structure of bone, from the exterior, through the medullary cavity, and the cancelli and Haversian canals, which are prolongations of it. Mr. Goodsir's views as to the nature of absorption derive powerful support from the circumstances under which that process takes place in cartilage ; for it is now well known, that this tissue is for the most part destitute of blood-vessels, only possessing them when its mass is bulky, or when it is undergoing change into bone. "It is evident, therefore, that in the process of ulceration in cartilage, it cannot be the usual blood-vessels of the part, which are the active agents. Still less likely is it that lymphatics, the existence of which has never been asserted in this texture, are the absorbing instruments." The following are, according to Mr. Goodsir, the appearances presented by a thin section made at right angles through the articular cartilage of a joint, at any part where it is covered by gelatinous membrane in scrofulous disease, or by false membrane in simple inflammatory conditions of the joint :

"On one edge of the section is the cartilage unaltered, with its corpuscles natural in position and size. On the opposite edge is the gelatinous, or false membrane, both consisting essentially of nucleated particles, intermixed, especially in the latter, with fibres and blood-vessels; and, in the former, with tubercular granular matter. In the immediate vicinity, and on both sides of the irregular edge of the section of cartilage, where it is connected to the membrane, certain remarkable appearances are seen. These consist, on the side of the cartilage, of a change in the shape and size of the cartilage-corpuscles. Instead of being of their usual form, they are larger, rounded, or oviform ; and instead of two or three nucleated cells in their interior, contain a mass of them. At the very edge of the ulcerated cartilage, the cellular contents of the enlarged cartilage-corpuscles communicate with the diseased membrane by openings more or less extended. Some of the ovoidal masses in the enlarged corpuscles may be seen half released from their cavities by the removal of the cartilage; and others of them may be observed in the substance of the false membrane, close to the cartilage, where they have been left by the entire removal of the cartilage which originally surrounded them.—If a portion of the false membrane be gradually torn off the cartilage, the latter will appear rough and honeycombed. Into each depression on its surface, a nipple-like projection of the false membrane penetrates. The cavities of the enlarged corpuscles of the cartilage open on the ulcerated surface by orifices of a size proportional to the extent of absorption of the walls of the corpuscle, and of the free surface of the cartilage.—The texture of the cartilage does not exhibit, during the progress of the ulceration, any trace of vascularity. The false membrane is vascular, and loops of capillary vessels dip into the substance of the nipple-like projections which fill the depressions on the ulcerated surface of the cartilage; but, with the exception of the enlargement of the corpuscles, and the peculiar development of their contents, no change has occurred in it. A layer of nucleated particles always exists between the loops of capillaries and the ulcerated surface. The cartilage, where it is not covered by the false membrane, is unchanged in structure. The membrane generally adheres with some firmness to the ulcerating surface ; in other instances it is loosely applied to it; but in all, the latter is accurately moulded to the former." (pp. 18-19.)

Sometimes, especially in scrofulous disease, the cartilage is attacked on its attached as well as on its free surface; and the process is essentially the same in the former case as in the latter, the vascular membrane with its nipple-shaped processes being derived from the tissue of the bone.

The fifth Essay, on "Secreting structures," is nearly identical with a paper which was published by Mr. Goodsir in the 'Transactions of the Royal Society of Edinburgh' for the year 1842, and which was noticed by us very shortly afterwards, (see Vol. XIV, p. 164.) We need not, therefore, enter into any detailed analysis of it; but shall content ourselves with noticing the modifications which it has undergone, subsequently to its first publication. It will be remembered by such of our readers as are conversant with the recent history of physiology, that a hypothesis was announced, some years since, by Purkinje, assigning to the nucleated epithelium of the gland-ducts the chief agency in the secretory function; but he made no statement to show that he had verified this hypothesis by observation. The reception of it as a part of our present system of belief is founded upon the observations of others; among whom Mr. Goodsir holds a very conspicuous place. Mr. Bowman was seeking, in the structure and functions of the human body, for a corroboration of this theory, whilst Mr. Goodsir was collecting a large amount of similar evidence from the ample stores furnished by comparative anatomy. In the original Memoir, the opinion was expressed, that as the nucleus is the reproductive organ of the cell, it has no direct concern in secretion, and that the cell-wall is the real instrument in the operation. Subsequent inquiry, however, has induced Mr. Goodsir to change that opinion; and we now find him advancing the doctrine, which had been independently advanced by Mr. Simon on other grounds, (see our last Number, p. 164,) that the *secretion of the cell is a product of the nucleus.*

"Since the publication of my paper on the secreting structures, in the Transactions of the Royal Society of Edinburgh, in 1842, I have satisfied myself that I was in error, in attributing to the cell-wall the important function of separating and preparing the secretion contained in the cell-cavity. The nucleus is the part which effects this. The secretion contained in the cavity of the cell appears to be the product of the solution of successive developments of the nucleus, which in some instances contains in its component vesicles the peculiar secretion, as in the bile-cells of certain mollusca, and in others becomes developed into the secretion itself, as in the seminal cells. In every instance, the nucleus is directed towards the source of nutritive matter, the cell-wall is opposed to the cavity into which the secretion is cast. This accords with that most important observation of Dr. Martin Barry, on the function of the nucleus in cellular development." (p. 33.)

Mr. Goodsir's observations, however, not only go to confirm the general doctrine of secretion by cells; they also throw important light upon the minute structure and development of glands. He shows that there are at least two different modes in which the secreting cells may be produced and arranged; viz. in *acini*, and in *follicles.* In the former case, the history of which he has particularly investigated in the testicle of squalus cornubicus, during the period of sexual vigour, the following appear to be the leading features of the process of development. A single nucleated cell is seen attached to the side of the duct, and causing a protrusion of its outer membrane;—this enlarges and projects more, and is seen to contain a few young cells grouped in a mass within it;—by a further enlargement it is removed from the duct, but remains connected with it by a hollow pedicle, being pyriform in shape, closed, and filled with nucleated cells;—and when its contents have arrived at maturity, the original parent-cell (now become merely the covering of the acinus, or collection of secreting cells) either dissolves away or discharges its contents through the pedicle into the duct. There is, consequently, in glands of this type, a continual development of new acini, which originate in points termed by Mr. Goodsir the "germinal spots of the gland." In glands of the follicular type, the early history may perhaps be the same as in the preceding case; but instead of the functions of the primary cell being brought to a close when its first crop of secreting cells is matured, it remains in a constant state of development and growth. Thus in the liver of *carcinus mœnas,* and other crustacea, each of the follicles may be observed to possess the following structure:

"The blind extremity of the follicle is slightly pointed, and contains in its interior a mass of perfectly transparent nucleated cells. From the blind extremity downwards, these cells appear in progressive states of development. At first they are mere primitive nucleated cells; further on they contain young cells; and beyond this they assume the characters of primary secreting cells, being distended with yellow bile, in which float oil-globules; the oil in some instances occupying the whole cell. Near the attached extremity of the follicle, an irregular passage exists in the midst of the cells, and allows the contents of the cells which bound it to pass on to the branches of the hepatic duct." (p. 30.)

Here it appears almost indubitable, that the development of the secreting cells takes place from a "germinal spot," situated at the blind extremity of the follicle. But in other glands, in which the follicles are greatly elongated so as to form tubes,—as in the human kidney,—Mr. Goodsir believes that the "germinal spots" are more uniformly diffused over the whole internal surface of the membranous wall. We should rather be inclined to say that there is no "germinal spot" at all; since in our apprehension this spot consists of an aggregation, in one point, of reproductive granules which are elsewhere separated and diffused. A similar variation is seen in numerous other instances;—certain vegetable cells having a distinct nucleus, cytoblast, or germinal spot; whilst others have the reproductive granules scattered through their interior; and even the germinal vesicle of animals, of which the germinal spot is usually so characteristic a feature, occasionally presents this latter condition.

But it would seem that, in the follicular glands, there is not only a successive development of secreting cells within the follicle, but the wall of the follicle itself is in a state of progressive growth; acquiring additions to its length at its blind extremity, and becoming absorbed at its attached extremity.

"Mr. H. S. Goodsir, in a paper on the development and metamorphoses of Caligus, read in the Wernerian Society, April 1842, has stated that the wall of the elongated and convoluted follicle, which constitutes the ovary in that genus, grows from its blind to its free extremity, at the same rate as the eggs advance in development and position. A progressive growth of this kind would account for the steady advance of its attached contents, and would also place the wall of the follicle in the same category with the primary vesicle, germinal membrane, or wall of the acinus, in the vesicular glands." (p. 31.)

Bringing together under one category, as we think he had a perfect right to do, the ordinary process of secretion, and the production of spermatozoa,—as taking place under conditions precisely analogous,—Mr. Goodsir had classified the products of the secreting action under the three following heads:—1st, the matter formed in the primary secreting cell-cavities;—2d, a mixture of fluid formed in these cell-cavities with the developed or undeveloped nuclei of the cells themselves;—and 3d,—a number of secondary cells passing out entire. He has since had an opportunity of verifying, and to an extent which he did not fully anticipate, the remarkable vital properties of the secretions of the third order just referred to.

"The distinctive character of these secretions is, that, when thrown into the cavity of the gland, they consist of entire cells, instead of being the result of the partial or entire dissolution of the secreting cells. It is the most remarkable peculiarity of this order of secretions, that, after the secreting cells have been separated from the gland, and cast into the duct or cavity, and therefore no longer a component part of the organism, they retain so much individuality of life, as to proceed in their development to a greater or less extent in their course along the canals or duct, before they arrive at their full extent of elimination.—The most remarkable instance of this peculiarity of secretions of this order, is that discovered by my brother, and recorded by him in a succeeding chapter. He has observed that the seminal secretion of the decapodous crustaceans undergoes successive developments in its course down the testis, but that it only becomes developed into spermatozoa after coitus, and in the spermotheca of the female. He has also ascertained that, apparently for the nourishment of the component cells of a secretion of this kind, a quantity of albuminous matter floats among them, by absorbing which they derive materials for development, after separation from the walls of the gland.—This albuminous matter he compares to the substance which, according to Dr. Martin Barry's researches, results from the secretion of certain cells of a brood, and affords nourishment to their survivors. It is one of several instances in which cells do not derive their nourishment from the blood, but from parts in their neighbourhood which have undergone solution; and it involves a principle which serves to explain many processes in health and disease." (p. 34.)

In Mr. H. S. Goodsir's paper, "On the testis and its secretion in the Decapodous Crustaceans," a great variety of curious facts are stated as the result of the author's examination of the subject; but as the one quoted in the preceding extract is the most important in a physiological point of view, we shall pass on to the next essay, "On the structure of the serous membranes," by Mr. John Goodsir. He agrees with Mr. Bowman as to the existence of a layer of basement membrane, between the single layer of epithelial cells and the vascular sub-serous areolar tissue. But he considers the basement membrane, here as elsewhere, as itself consisting of flattened cells, with persistent nuclei, which give it the character of a "germinal membrane."

"The germinal membrane does not in general show the lines of junction of its component flattened cells. These appear to be elongated in the form of ribands; their nuclei, or the germinal spots of the membrane, being elongated, expanded at one extremity, pointed at the other, and somewhat bent upon themselves. The direction of these flattened cells and nuclei is the same in any one part of the membrane, this direction being in general parallel to the subjacent blood-vessels, in the direction of which they exist in the greatest numbers. The germinal spots are bright and crystalline, and may, or may not, according to their condition, contain smaller cells in their interior. They are not to be confounded with the fibres of the areolar texture, or with elastic filaments, or with the nuclei of the capillary vessels of the subserous texture, or with paler, ovoidal, somewhat indistinct cells, scattered throughout that texture, and which appear to be connected with the common areolar fibres. These flattened riband-shaped scales, and bright crystalline nuclei, which form the germinal or basement membrane of the serous coat, appear to be identical with the objects described by Valentin, Pappenheim, and Henle, and named by the latter nucleated fibres." (p. 42.)

The following remarks fully coincide with the ideas that we had independently formed, on the curious point to which they relate :

"I have been in the habit of considering the highly vascular fringes and processes of the synovial membranes as more active in the formation of epithelium, and therefore more closely allied to the secreting organs, than other portions of these membranes. If this be the case, Clopton Havers was not mistaken in his ideas regarding the functions of these vascular fringes. They are situated where they cannot interfere with the motions of the joint. They hang into those parts of the cavity best fitted for containing and acting as reservoirs of synovia; and their high vascularity, and the pulpy nature of their serous covering, tend to strengthen this opinion." (p. 43.)

With the following opinions, also, on the subject of inflammation, we need scarcely say that we heartily concur :

"The phenomena attending inflammatory action of the membranes are highly interesting. The capillaries are all on one side of the membrane, and yet the serum and lymph are on the other. The capillary vessels in healthy action have no power in themselves of throwing out any of their contents. They do not secrete in virtue of any power inherent in themselves. Do they acquire this power during inflammation ? Or will any of the hypotheses of effusion account for the lymph and serum being on the free surface of the serous membranes, and so little, if any, in the subserous textures ?

"I do not see how we can, in the present state of the science, account for phenomena of this kind, by referring them to actions of the extreme vessels. We must look for an explanation, I am inclined to believe, in a disturbance of the forces which naturally exist in the extra-vascular portions of the inflamed part." (p. 43.)

The short paper which succeeds, on the "Structure of the lymphatic glands," by Mr. John Goodsir, is of peculiar interest. His account of the mode of communication between the afferent and efferent lymphatics, in these curious bodies, does not differ much from that usually given. In the lymphatic glands of the human subject, he considers that the terminal branches of the afferent vessels form a network more or less dense with the radicles of the efferent; and he regards the cells, which not unfrequently present themselves in glands that have been injected with mercury, as resulting from the over-distension of short vessels. But in some of the lower mammals, it appears that there is a partial or entire obliteration of some of the meshes ; so as to produce cavities more or less extended, with bars or threads passing from wall to wall, the lymphatics opening into them. Of the three tunics possessed by the extra-glandular lymphatics, the outer one is lost as soon as they enter the gland ; passing over its surface, so as to form a capsule ; and investing the lymphatics again, at their departure from it. The middle or fibrous tunic disappears less suddenly ; being sufficiently apparent on the lymphatics near the surface of the gland ; but being met with sparingly towards the centre. It is in the account given by Mr. Goodsir of the internal tunic and epithelium of the intra-glandular lymphatic network, that the novelty of his observations consists.

"In the short dilated anastomosing branches, which form the intra-glandular network, this tunic has become so thin and opaque, that the vessels will no longer transmit the light, and appear as if they were stuffed full of a granular matter. When these thickened and dilated vessels are cut, torn, or broken, so as to display their structure, it may be observed that two parts enter into their composition; an extremely fine external membrane, and a thick granular substance which lines the membrane.—The external membrane is extremely thin and transparent. In its substance there are arranged, at regular distances, ovoidal bodies, so placed that their long diameters are all in the same direction. The distance of these bodies from one another is somewhat greater than their long diameters. They are imbedded in the substance and form part of the membrane. They are hollow, and contain one or more rounded vesicles grouped together in their interior. I have seen portions of this membrane after it has been acted on by acetic acid, present an appearance of being broken up into flat semi-transparent scales, united by their edges, each scale consisting of one of the nucleated ovoidal bodies, and a portion of the surrounding membrane.

"The thick granular substance, which is attached to the internal surface of the membrane just described, is composed entirely of nucleated particles, closely packed together, and cohering to one another. The thickness of this layer of granular substance is so considerable, as to render the vessel of which it is a part almost opaque, encroaching on its cavity, and leaving a comparatively narrow canal for the passage of the lymph and chyle. This canal appears to be somewhat irregular, in consequence of the greater exuberance of the granular substance in some spots, and its deficiency in others. This circumstance also accounts for the greater transparency of the vessels at certain parts of their extent. The canal is not lined by a membrane, but appears to me to be irregularly pierced through the granular substance: the projections and hollows of which, as well as the superficial layer of its nucleated particles, are freely bathed by the lymph and the chyle.—The nucleated particles are on an average about the 5000th of an inch in diameter. They are spherical, and contain a nucleus, which consists of one or more particles. Their walls are very distinct, especially after being treated with acetic acid, which reduces their size somewhat, without dissolving or breaking them up.—The layer of particles which has now been described is thickest in lymphatics towards the centre of the gland. If it be examined in either direction towards the afferent or efferent branches, it will be found to become thinner, and, at last, to be continuous with the layer of flat epithelium-scales of the extra-glandular lymphatics." (p. 47.)

We can scarcely doubt that Mr. Goodsir is correct in regarding the spots in the primary membrane in the light of "germinal spots," developing the layers of cells that lie upon it. The large number of such cells that may be found in the fluid squeezed from the lymphatic glands when cut across, far exceeding, in their proportion to the fluid, those of either the afferent or efferent lymphatics, as was pointed out some time since by Mr. Gulliver,—seems to indicate that a continual production of them must be taking place in those situations; whilst the continuity of the mass which they form in the interior of the gland, with the epithelium-layer of the external lymphatic trunks, shows that their character must be essentially the same. Hence we may regard the lymphatic glands as possessing the *operative* portion of the lymphatic vessels,—namely, the basement membrane and the epithelial layer,—in a very concentrated form; and this is precisely the view to which we should be led, by the consideration of the facts of comparative anatomy on the larger scale. For the lymphatic system, first introduced in the class of fishes, attains its greatest development as to *length* in reptiles; but it possesses very few glands. In birds and mammals, the apparent development of the lymphatic system is not so great; but its real development is far greater; since what is wanting in length is more than made up for in concentration. It is well known that a considerable change takes place in the nature of the absorbed fluid, during its passage along the lacteal and lymphatic trunks; and it can now, we think, be hardly doubted, that the cells lining the lymphatic vessels, but especially those so abundantly present in the glands, are the real agents in producing this change. That the term "glands" applied to these bodies, is consequently not so incorrect as might be thought from their structure, appears from the preceding considerations, and also from the following remarks of Mr. Goodsir, in regard to the arrangement of their vessels.

"The description usually given of the arrangement of the blood-vessels in the lymphatic glands, is sufficiently correct. The ultimate capillaries, as I have observed, do not ramify in the substance of the germinal membrane of the intra-glandular lymphatics; but are merely in contact with its external surface. In this respect they resemble the ultimate ducts of the true secreting glands.—The capillary network which surrounds the intra-glandular lymphatics, is as fine as that which supplies the ultimate secreting ducts; and has the same purpose in both,—to afford matter for the continued formation of secreting epithelium on the internal surface of the germinal membrane." (p. 48.)

There is this difference, however, which Mr. Goodsir seems to have overlooked;—that the blood distributed to ordinary glands has to furnish, not only the materials for the production of the epithelial or secreting cells, but also the materials of the secreted product;—whilst in the case of the lymphatic ganglia, the function of the vessels is only to supply the former kind of materials, the latter being furnished by the afferent lymphatics.

The next essay, on the "Structure of the human Placenta," is the most elaborate and complete of the whole series; and it appears to us to leave but little to be made out by future investigations in regard to the minute conformation of this important organ, and the connexion of the mother and fœtus. The chief additions to our knowledge that may be expected in regard to it, will probably be derived more from the investigation of its comparative structure in other mammals, than from the continued prosecution of minute inquiries as to its own. In order to place our readers on a level with the subject, at the point at which it was taken up by Mr. Goodsir, we shall briefly state the facts which had been ascertained by Professors Weber and Reid, in regard to the character of the human placenta, and the relation of its fœtal and maternal portions. The *fœtal* portion consists of an immense number of villi or minute vascillary tufts, which contain the capillaries that form the terminations of the umbilical artery, and the radicles of the umbilical vein. These tufts hang down, as it were, in the cavity of the placenta; which belongs to the maternal portion of the organ, and which may be regarded as a vast sinus, receiving its blood from the curling arteries of the uterus, and returning it to the large veins of that viscus. The fœtal tufts do not hang freely in this cavity, however; but they are bound in their places by reflexions of its lining membrane, which closely enwrap them, and form a series of partitions, crossing the placental cavity in every direction, and dividing it into a series of cells that communicate freely with each other. The mode in which the villi are thus kept *without* the cavity or placental sinus, whilst occupying so large a part of its contained space, is precisely that in which the abdominal viscera occupy the abdominal cavity, whilst still external to the peritoneal sac, and bound down by the reflexions of that membrane. As there is no more direct communication between the vascular systems of the mother and fœtus than this, it is evident that the whole change which the blood of the latter undergoes through the agency of that of the former, must be effected through the medium of the villi, and the lining membrane of the placental sac which covers them. These villi, therefore, serve two purposes; having for their object both the absorption of fresh nutriment, like the intestinal villi; and also the aeration of the fœtal blood which passes through them, as in the gills of aquatic animals.

We now proceed to notice the inquiries of Mr. Goodsir into the minute structure of the parts whose general arrangement we have indicated; and we cannot do justice to them without making large quotations from his essay. The first relate to the structure of the tufts and villi of the placenta:

"A placental tuft resembles a tree. It consists of a trunk, of primary branches, and of secondary branches or terminal villi, which are attached as solitary villi to the sides of the primary branches, and to the extremities of the latter, in which case they frequently present a digitated arrangement.......The digitated villi are only solitary villi grouped together at the extremity of a primary branch.

"The trunk, the primary branches, and the terminal villi of the tufts, are covered by a very fine transparent membrane, apparently devoid of any structure. This membrane may be described as bounding the whole tuft, passing from the trunk to the branches, and from these to the villi, the free extremities of which it closely covers Its free surface is smooth and glistening; its attached surface is somewhat rough.

"Immediately under the membrane just described is a layer of cells. They are flattened spheroids, slightly quadrilateral in outline, from the manner in which they are packed together. When a tuft is viewed in profile, under compression, its edges exhibit the appearance of a double line, which leads the observer to suppose that its bounding membrane is double, with the cells just described situated between the two laminæ. In the space between the two lines, the nuclei of the cells may be seen in the form of dark oval spots, and the septa formed by the walls of contiguous cells are also visible. At variable distances, the space between the two lines widens out into a triangular form, the base towards the external membrane, the apex towards the centre of the villus. The wider space is produced by a larger group of cells, which appear to be passing off from a spot in the centre of the mass. The groups of cells I am now describing are germinal spots. They are the centres from which new cells are constantly passing off, to supply the loss of those which have disappeared in the performance of their important function." (p. 51.)

This *external* membrane of the fœtal tufts, and the layer of cells generated from it, will be presently shown to be a part of the *maternal* structure; and to be in fact a continuation of the lining membrane of the uterus. We have now to notice the *internal* membrane, which is the boundary of the *fœtal* portion; and another set of cells, belonging to the latter, and analogous to those of the intestinal villi.

"When a villus, under gentle compression, is viewed by transmitted light, there is perceived under the structures already described, and immediately bounding the blood-vessels and other parts to be afterwards examined, a membrane finer and more transparent than the external membrane, but strong and firm in its texture. This membrane is most distinctly seen when it passes from one loop or coil of the blood-vessel of the villus on to another. It separates very easily from the internal surface of the layer of external cells. I am not disposed to believe that it is attached to this layer, but am of opinion that the spaces which frequently exist between them, even in villi which have undergone no violence, are due to the presence of a fluid matter, the nature of which will be afterwards considered. Be this as it may, pressure very easily separates this membrane from the external cells; the latter invariably remaining attached to the external membrane, the former continuing in every instance closely rolled round the internal structures of the villus, and following them in all their changes of position." (p. 52.)

Mr. Goodsir then gives an elaborate account of the distribution of the vessels in the villi; through which, however, we need not follow him, as the main facts had been previously ascertained by Professor Weber and Reid, and Mr. Dalrymple. The umbilical artery terminates in vessels, which enter the villi, proceed to their extremities, and return as the radicles of veins,—the same vessel, however, sometimes entering and returning from two or more villi, before it becomes continuous with a vein. "This species of blood-vessel, although it cannot be considered as either artery or vein, cannot nevertheless be denominated, in precise anatomical language, a capillary. It differs from artery and vein in retaining throughout the same mean diameter; and from the capillary, properly so called, in its greater caliber, containing four or six blood-discs abreast. It is also peculiar in exhibiting sudden constrictions and dilatations, like an intestine. These changes in form are most remarkable at the spots where the vessel makes sudden turns, coils, or convolutions."—We have now to notice an important discovery of Mr. Goodsir's; that of the *internal cells* of the villus, which are precisely analogous to those of the intestinal villi.

"Within the internal membrane, and on the external surface of the umbilical capillaries, are cells which I have named the internal cells of the tuft. When the vessels are engorged, these cells are seen with difficulty. When the vessels are moderately distended, and the internal membrane separated from the external cells by moderate pressure, the cells now under consideration come into view. They are best seen in the spaces left between the internal membrane and the retiring angles formed by the coils or loops of the vessels, and in the vacant spaces formed by these loops. These cells are egg-shaped, highly transparent, and are defined by the instrument with difficulty; but their nuclei are easily perceived. They appear to be filled with a highly transparent refractive matter. This system of cells fills the whole space which intervenes between the internal membrane of the villus and the vessels; and gives to this part of the organ a mottled appearance." (p. 54.)

We shall anticipate the subsequent part of the paper, by here stating his conclusions in regard to the nature and offices of these several structures; in which we fully accord with him:

"1. The placenta, as has long been admitted, consists of a fœtal and of a maternal portion intermixed. But the maternal portion, instead of consisting of a part of the vascular system of the mother only, includes the whole of the external cells of the villi.

"2. The external membrane of the placental villi is a portion of the wall of the vascular system of the mother, continuous with the rest of that wall, through the medium of the placental threads, and lining membrane of the placental cavity.

"3. The system of the external cells of the placental villi belongs to the decidua, and is continuous with the parietal division through the medium of the cavities of the placental threads. This portion of the decidua has been named the central division of the placental decidua, and the threads decidual bars.

"4. The function of the external cells of the placental villi, is to separate from the blood of the mother the matter destined for the blood of the fœtus. They are, therefore, secreting cells, and are the remains of the secreting mucous membrane of the uterus.

"5. Immediately within the external cells of the placental villi, there is a membrane which I have named the internal membrane of the villi. This membrane belongs to the system of the fœtus, and is the external or bounding membrane of the villi of the chorion.

"6. Inclosed within the internal membrane of the placental villi, is a system of cells, which belong to the system of the fœtus, and are the cells of the villi of the chorion. These are the internal cells of the placental villus.

"7. The function of the internal cells of the placental villi is to absorb through the internal membrane the matter secreted by the agency of the external cells of the villi.

"8. The external cells of the placental villi perform, during intra-uterine existence, a function for which is substituted in extra-uterine life, the digestive action of the gastro-intestinal mucous membrane.

"9. The internal cells of the placental villi perform during intra-uterine existence a function, for which is substituted in extra-uterine life, the action of the absorbing chyle-cells of the intestinal villi.

"10. The placenta, therefore, not only performs, as has been always admitted, the function of a lung, but also the function of an intestinal tube." (p. 63.)

From Mr. Goodsir's full description of the villi of the chorion, previously to their becoming vascular, we abridge the following account. The substance of the tufts consists of nucleated cells of various sizes, the smaller being situated in the interior of the larger and in the intermediate spaces. Their surface is bounded by a fine but very distinct membrane, which, when minutely examined, is seen to consist of flattened cells united by their edges. The free extremity of each villus of the tuft is bulbous. The cells which constitute this swelling are arranged round a central spot,—the germinal spot of the villus; from which new cells are continually arising, that produce, by their development the elongation of the villus. A similar enlargement may be occasionally seen on the sides of the stems; and each of these spots is the commencement of a new villus or stem, which, as it elongates, carries forward on its extremities the swelling from which it arose. Blood-vessels first appear in the villi, when the allantois reaches and applies itself to a certain portion of the internal surface of the chorion. The umbilical vessels then communicate with the substance of the villi, and become continuous with loops in their interior. Those villi in which the blood-vessels do not undergo any further development, as the ovum increases in size, become more widely separated, and lose their purpose in the economy. The villi, again, in which vessels form, in connexion with the umbilical vessels, increase in number; as their vessels enlarge, the cells diminish, but they are always found surrounding the terminal loop of vessels in the situation of the germinal spot; and the bounding membrane of the villus coalesces with the contained cells along the stems and branches of the villi, so as to form a fibrous texture, whilst it remains distinct at their extremities. Thus is accomplished the metamorphosis of the villus of the chorion into the placental tuft.

We come, lastly, to Mr. Goodsir's investigation of the structure and formation of the maternal portion of the placenta; a chief element of which relates to that *quæstio vexata* of embryology, the formation of the decidua. By the observations of Professors Weber and Sharpey it had been ascertained, that when impregnation has taken place, the mucous membrane of the uterus swells and becomes lax, its follicles increase in size and secrete a granular matter, and its capillaries enlarge in a proportionate degree. Mr. Goodsir has remarked, however, that the inter-follicular spaces, in which the network of capillaries lies, are occupied by a texture consisting entirely of nucleated particles; "this is a tissue represented by Baer and Wagner, described by them as surrounding what they supposed to be uterine papillæ (really the enlarged follicles), and considered by them as decidua." The increased thickness of the mucous membrane appears as much due to the development of this inter-follicular substance, as to the enlargement of the follicles.

"About the time at which the ovum reaches the uterus, the developed mucous membrane or decidua begins to secrete; the os uteri becomes plugged up by this secretion, where it assumes the form of elongated epithelial cells; the cavity of the uterus becomes filled with a fluid secretion, the 'hydroperione' of Breschet; and in the immediate neighbourhood of the ovum, the secretion consists of cells of a spherical form. The cells which are separated in the neighbourhood of the ovum, I consider as a secretion of the third order. They have passed off from the uterine glands entire, and possess a power peculiar to the third order of secretions, the power of undergoing further development after being detached from the germinal spots or membrane of the secreting organ.

"From what has now been stated, it appears that the decidua consists of two distinct elements; the mucous membrane of the uterus thickened by a peculiar development, and a non-vascular cellular substance, the product of the uterine follicles. The former constitutes at a later period the greater part of the decidua vera; the latter, the decidua reflexa. This view of the constitution of the decidua clears up the doubts which were entertained regarding the arrangement of these membranes at the os uteri and entrances of the Fallopian tubes. It is evident that these orifices will be open or closed, just as the cellular secretion is more or less plentiful, or in a state of more or less vigorous development. It also removes the difficulty of explaining how the decidua covers the ovum; a difficulty which cannot be reconciled with the views of Dr. Sharpey, who is obliged to suppose the deposition of lymph, which is only the old view of the constitution of the decidua.

"When the ovum enters the cavity of the uterus, the cellular decidua surrounds it, and becomes what has been named the decidua reflexa, by a continuation of the same action by which it had been increasing in quantity before the arrival of the ovum. The cellular decidua grows around the ovum by the formation of new cells, the product of those in whose vicinity the ovum happens to be situated." (p. 59.)

At this stage of its growth, the ovum with its external membrane, the chorion, which is covered by the tufts whose structure and functions have been just described, is imbedded in a substance that consists entirely of active nucleated cells. The absorbing cells of the tufts are constantly taking up either the matter resulting from the solution of the cells of the cellular decidua, or the fluid contained in those cells;—in either case, from matter supplied by the vessels of the uterus, but selected and prepared by the cells of its lining membrane, now become the decidua. Fully coinciding thus far with Mr. Goodsir, we cannot agree with him that "the cellular decidua represents in the gestation of the mammal the albumen of the egg of the oviparous animal." For though they are both supplied by a certain portion of the oviduct, and are both brought into play after the nourishment supplied by the ovum is exhausted, yet there is this important difference in their respective situations,—that the cellular decidua is a formation *external* to the chorion,—whilst the albumen of the bird's egg is deposited *within* that fibrous membrane, which lines the shell and forms its organic basis, and which must be regarded as the representative of the chorion of mammals. The true analogue of the albumen of the bird's egg, we take to be the fluid, which is found between the zona pellucida or vitelline membrane of mammals, and the chorion, soon after the formation of the latter. This, too, is secreted by the oviduct, and appears to serve a purpose in the temporary nutrition of the ovum. We regard Mr. Goodsir as more correct in the following analogy. "I have also been in the habit of considering the uterine cotyledons of the ruminant and other mammalia as a permanent decidua vera, and the milky secretion interposed between them and the fœtal cotyledons as decidua reflexa in its primitive and simplest form." (p. 59.)

The following is Mr. Goodsir's account of the incipient formation of the placenta, out of the elements supplied by the vascular villi of the chorion on the one hand, and the decidua on the other:

"The vessels of the decidua enlarge, and assume the appearance of sinuses, encroaching on the space formerly occupied by the cellular decidua; in the midst of which the villi of the chorion are imbedded. This increase in the caliber of the decidual capillaries goes on to such an extent, that finally the villi are completely bound up and covered by the membrane which constitutes the walls of the vessels, this membrane following the contour of all the villi, and even passing to a certain extent over the branches and stems of the tufts. Between this membrane, or wall of the enlarged decidual vessels, and the internal membrane of the villi, there still remains a layer of the cells of the decidua.

"From this period, up to the full time, all that portion of decidua in connexion with the group of enlarged capillaries, and vascular tufts of the chorion, and which may now be called a placenta, is divided into two portions. The first portion of the decidua in connexion with the placenta, or forming a part of it, is situated between that organ and the wall of the uterus. This is the only portion of the placental decidua, with which anatomists have been hitherto acquainted; and I shall name it the parietal portion. It has a gelatinous appearance, and consists of rounded or oval cells. Two sets of vessels pass into it from the uterus. The first set includes vessels of large size, which pass through it for the purpose of supplying the placenta with maternal blood for the use of the fœtus. These may be named the maternal functional vessels of the placenta. The second set are capillary vessels, and pass into this portion of the decidua for the purpose of nourishing it. They are the nutritive vessels of the placenta." (p. 60.)

By the careful dissection of an unopened uterus at the full time, in the manner formerly adopted by Mr. Owen, Mr. Goodsir has satisfied himself that the veins of that organ form many anastomoses near its internal surface; the spaces which they inclose presenting the appearance of narrow flat bands. When the venous membrane is traced in its passage from the uterus into the placenta, it passes suddenly to each side to line the great cavity of that organ; the flat bands just mentioned become smaller; and on entering the cavity itself, the bands are seen to assume the appearance of threads, which pass in great numbers from the edges of the venous openings, and from the walls of the cavity of the placenta, on to the extremities and sides of the villi and tufts of the placenta. The whole mass of tufts is connected together by similar threads, and is attached in like manner to that surface of the parietal decidua which is covered by the venous membrane. On minute examination, Mr. Goodsir found these threads to be tubular; and the membrane which they formed was seen to be continuous in one direction with the lining membrane of the vascular system of the mother, and in the other with the external membrane of the tufts of the placenta. These threads, as well as their cavities, were somewhat funnel-shaped at each extremity; and the funnel-shaped portions of the cavities of the threads, as well as, in some instances, the whole length of the tube, were found to be full of cells, which were continuous in the one direction with the parietal decidua of the placenta, and in the other with the external cells of the placental villi. This account of the appearances actually observed in the dissection of a uterus and placenta, fully bears out in our estimation the statements we have previously quoted with regard to the relations of the different structures.

We have dwelt the longer on this valuable paper, because we consider the elucidation of the structure and formation of the placenta given by Mr. Goodsir, as alike valuable in itself and creditable to him; since it is evident that no previous observer, among the many who have devoted a large amount of time and attention to the subject, at all approaches him in the exactness and completeness of his analysis. The formation and offices of the decidua reflexa strike us as the portion of his researches which leaves most room for further investigation; and this we have no doubt that he will bestow upon the subject, as opportunity presents itself. We cannot forbear noticing, that the analysis of this complex organ—the placenta,—and the determination of the real nature and connexions of its different parts, has been effected entirely through the aid of the microscope; and that to the use of this instrument also we owe the settlement of the long-disputed question, in regard to the mode in which the elements of the maternal blood find their way into the fœtal system.

Two papers by Mr. John Goodsir,—on the structure and economy of bone, and on the mode of reproduction after death of the shaft of a long bone,—conclude his portion of the work. The former of these commences with the following general remarks, which cannot in our apprehension be kept too strongly in view, by those who devote themselves to the study of general anatomy and physiology.

"A texture may be considered either by itself, or in connexion with the parts which usually accompany it. These subsidiary parts may be entirely removed, without interfering with the anatomical constitution of the texture. It is essentially non-vascular; neither vessels nor nerves entering into its intimate structure. It possesses in itself those powers by which it is nourished, produces its kind, and performs the actions for which it is destined; the subsidiary or superadded parts supplying it with materials which it appropriates by its own inherent powers, or connecting it in sympathetic and harmonious action with other parts of the organism to which it belongs.—In none of the textures are these characters more distinctly seen than in the osseous. A well-macerated bone is one of the most easily made, and at the same time one of the most curious, of anatomical preparations. It is a perfect example of a texture completely isolated; the vessels, nerves, membranes, and fat, are all separated; and nothing is left but the non-vascular osseous substance." (p. 64.)

We shall not follow Mr. Goodsir through his account of the structure of bone, since it adds little to what was previously known. His attention is peculiarly directed to the inquiry into the offices of the periosteum; and he concludes, most justly as we think, that it is a structure far less important in the nutrition of bone, than the membrane lining the Haversian canals and cancelli. The following observations we believe to be partly new; the cells alluded to have been described by Mr. Tomes as existing during the process of ossification; but he does not seem to have recognized them as permanent constituents of the bone.

"Between the blood-vessels and the walls of the Haversian canals, there is a layer of cellular substance; which is the product, its cells being the descendants of the corpuscles of the cartilage or matrix in which the bone was originally formed. It forms a blastema, originally produced round each cartilage-corpuscle by development into a linear series, perpendicular to the ossifying surface; each of the secondary cartilage-corpuscles remaining as centres, or sources of new centres, of nutrition of the future bone; their progeny forming the cellular mass, which becomes inclosed in the capsules of compact primary bone. When these capsules have opened into one another to form the Haversian canals,—a process similar to the mode of development of gland-ducts and capillaries,—the cellular mass surrounds the vessels in their course, and separates them from the walls of the canals.

"That this cellular layer plays an important part in the economy of bone, appears probable from the prominent position it holds in its development, and from the intimate connexion of the Haversian canals with all the morbid changes of bone. Its existence, great extent, and probable powers, cannot be overlooked in any question regarding the economy of bone in health and disease.

"The cellular mass, just described, fills the cancelli, or enlarged Haversian chambers, of fœtal bones; and in this situation has not been overlooked by former observers. In adult bones, it is in the medullary cavity, cancelli, and to a certain extent in the larger Haversian canals, replaced by fat-cells.

"On the surface of young and vigorous bones I have observed numerous cells, flattened, elongated, and more or less turgid, belonging doubtless to the system of Haversian cells." (p. 67.)

In the second of the two papers, whose titles have been just quoted, Mr. Goodsir enters more formally upon the question, of which, as is well known, the affirmative is strongly maintained by Professor Syme; "whether the periosteum possesses the power of forming new osseous substance, independently of any assistance from the bone itself." Mr. Goodsir's views of the nature of nutritive action would lead him, on *a priori* grounds, to question the possibility of the formation of bone by any action of the *vessels* of the periosteum or of any other membrane; and to maintain, upon the principle of "like producing like," that new bone can only be formed in continuity with previously existing osseous tissue. He has, with these views, instituted a careful examination of the experiments and observations relied on by Mr. Syme in support of his position; and he has arrived at the following conclusions in regard to them, from which the nature and direction of his inquiries may be inferred:

"As, therefore, it has been found impossible to separate the periosteum in living animals, without detaching shreds of bone along with it; as in necrosis of the shafts of long bones, portions of the old osseous texture may be detected in the periosteal sheath opposite ulcerations of the dead shaft; and as consistent with what is at present held regarding the powers of capillary vessels and the origin of the textures, we are compelled to assent to the doctrine, that periosteum does not possess an independent power of forming osseous substance.

"The participation of the periosteum in the office of regeneration—an important principle in surgery—is not denied in this conclusion." (p. 73.)

We cannot regard this question as completely settled, until the structure and origin of *adventitious* bony deposits in other tissues shall have been more fully elucidated, and shall have been compared with that of the supposed periosteal formation.

Two papers by Mr. Harry Goodsir occupy the remainder of the volume: the first being a short account of the mode of reproduction of lost parts in the crustacea; and the second, which is the longest in the collection, being devoted to the anatomy and development of the cystic entozoa. This last subject lies so very wide of the preceding, that we think it better not to touch upon it for the present; more especially as Mr. Goodsir's views differ so essentially from those of others who have recently attended to the subject, that we feel at present in considerable difficulty as to the real truth. But we shall briefly notice some of the points elucidated in the former paper, as being of general physiological interest.

It has long been known that all the species of crustacea have the power of regenerating parts of their limbs that have been accidentally lost; and that, in the species with which we are best acquainted, if one or more of the last phalanges of the leg be seriously injured, the animal, by an effort of its own, throws off the remaining parts of the limb, from a point near the basal extremity of the first phalanx. This it will also sometimes do, simply for the purpose of making its escape when held by its leg. After a time, a new limb sprouts from the cicatrix; and this gradually acquires the size and proportions of the old one. It appears that in the crab and lobster, and others of the higher decapods, this regeneration will *only* take place from the points just named; but according to Mr. Goodsir, among the lower crustacea the power of regeneration is more extended, as a limb broken off at any part of its phalanges will grow. The part of the first phalanx at which the separation takes place is much contracted for the length of half an inch or more, in the common edible crab; and it is described by Mr. H. Goodsir as being filled with a fibrous, gelatinous, glandular-looking mass,—the organ which supplies the germs for future limbs. When a thin transverse section of this organ is placed under the microscope, it presents, in the first place, a foramen for the transmission of the vessels and nerves; and its substance is divided into two parts by a thick annular fibrous-looking band, the substance within which is much more transparent than that on its exterior; the former consists of numerous small cells, all having nuclei or nucleoli within them, and suspended in a thickish transparent liquid; whilst the latter consists of a confused mass of large cells, filling up the whole space between the fibrous band and the membrane of the shell. This fibrous band belongs, according to Mr. H. Goodsir, to a very peculiar system of vessels, which are very generally distributed throughout the body of the animal, but the relations of which he has not yet been able to elucidate. The following is his account of the process of reproduction.

"Immediately on the limb being thrown off, a quantity of blood escapes, which is soon stopped by the retraction of the vessels. After this takes place, we see the small open foramen for the passage of the artery and nerve, which becomes closed almost immediately by means of a slight film which spreads over the whole of the exposed surface. When this surface is examined some hours after the loss, we find that the small cavity of the foramen is slightly filled up with a body resembling a nucleated cell. This cell is the germ of the future leg, and very shortly increases in size, so as gradually to push out the film alluded to above, which is now become a thick strong cicatrix. During the time that this is going on, the whole of the exposed surface becomes tense and bulging; but this gradually decreases round the circumference as the central nucleus increases in size, which it does at first longitudinally, and then transversely. As it increases in size, the cicatrix, which still surrounds it as a sac, becomes thinner and thinner, until it bursts; when the limb, which has hitherto been bent upon itself, becomes stretched out, and has all the appearance of a perfect limb, except in size.

"As far as my observations have yet gone, it appears to me that the germinal cell is derived from one of those which are nearest the central opening on the said surface. This cell follows the ordinary course of development, by the nucleus breaking up into nucleoli, which in time become parent-cells, each of which again undergoes the same process. This proceeds for several stages, all the less important cells dissolving and serving as nourishment for the more important ones, until the number of centres is reduced to five, the number of joints required; which, by a constant process of a similar nature, assume the form of the future leg." (p. 78.)

We would close as we began, by thanking the Messrs. Goodsir most earnestly in our own name, and in that of the profession, for their most valuable additions to our stock of knowledge. We wish that we could see the same activity and zeal displayed by some of those who hold the few situations in which a physiologist can make a living by the business of instruction. Upon such, it appears to us, the world has the chief claim for labours that may extend the domain of science; yet we too often see that those who attained the early distinction which procured them these posts, sink into inactivity as soon as they are attained.

We think it right to suggest to the Messrs. Goodsir, in conclusion, that, when they next bring their discoveries under the public eye, they procure the assistance of some friend, less versed perhaps in microscopy, but more in the craft of authorship, to revise their essays before they go to press. Were we disposed to be critical upon errors of style and grammar, we could have pointed to not a few, which deserve grave reprehension. Many of these seriously obscure the authors' meaning; and we have consequently thought it necessary to correct them, in making our quotations. We say no more, in consideration of the sterling value of the production.

---

